# Colorectal Polyp Image Detection and Classification through Grayscale Images and Deep Learning

**DOI:** 10.3390/s21185995

**Published:** 2021-09-07

**Authors:** Chen-Ming Hsu, Chien-Chang Hsu, Zhe-Ming Hsu, Feng-Yu Shih, Meng-Lin Chang, Tsung-Hsing Chen

**Affiliations:** 1 Department of Gastroenterology and Hepatology, Linkou Chang Gung Memorial Hospital and Chang Gung University College of Medicine, No. 5, Fuxing St., Guishan Dist., Taoyuan City 333, Taiwan; hsu3060e@cgmh.org.tw (C.-M.H.); q122583@cgmh.org.tw (T.-H.C.); 2 Department of Computer Science and Information Engineering, Fu-Jen Catholic University, 510 Chung Cheng Rd., Hsinchuang Dist., New Taipei City 242, Taiwan; 405261382@mail.fju.edu.tw (Z.-M.H.); 403261443@mail.fju.edu.tw (F.-Y.S.); 3 Graduate Institute of Applied Science and Engineering, Fu-Jen Catholic University, 510 Chung Cheng Rd., Hsinchuang Dist., New Taipei City 242, Taiwan; oro.tidyscoundrel@gmail.com

**Keywords:** colorectal polyp, grayscale image, colonoscopy, convolutional neural network, computer-assisted colorectal polyp analysis

## Abstract

Colonoscopy screening and colonoscopic polypectomy can decrease the incidence and mortality rate of colorectal cancer (CRC). The adenoma detection rate and accuracy of diagnosis of colorectal polyp which vary in different experienced endoscopists have impact on the colonoscopy protection effect of CRC. The work proposed a colorectal polyp image detection and classification system through grayscale images and deep learning. The system collected the data of CVC-Clinic and 1000 colorectal polyp images of Linkou Chang Gung Medical Hospital. The red-green-blue (RGB) images were transformed to 0 to 255 grayscale images. Polyp detection and classification were performed by convolutional neural network (CNN) model. Data for polyp detection was divided into five groups and tested by 5-fold validation. The accuracy of polyp detection was 95.1% for grayscale images which is higher than 94.1% for RGB and narrow-band images. The diagnostic accuracy, precision and recall rates were 82.8%, 82.5% and 95.2% for narrow-band images, respectively. The experimental results show that grayscale images achieve an equivalent or even higher accuracy of polyp detection than RGB images for lightweight computation. It is also found that the accuracy of polyp detection and classification is dramatically decrease when the size of polyp images small than 1600 pixels. It is recommended that clinicians could adjust the distance between the lens and polyps appropriately to enhance the system performance when conducting computer-assisted colorectal polyp analysis.

## 1. Introduction

Colorectal cancer is globally the third most prevalent cancer and mainly derived from pre-malignant colorectal polyps [1]. Colonoscopy screening and endoscopic resection of colorectal polyps can reduce the incidence and mortality of colorectal cancer [2]. Image-enhanced colonoscopy with contrast enhancement through dyes or optical strategies facilitates the detection and observation of colorectal lesions [3]. However, the accuracy of the detection and diagnosis of colorectal polyps varies depending on the experience of endoscopists, thus influencing the effectiveness of colonoscopy in reducing the incidence and mortality of colorectal cancer [3,4].

For detailed observation of the colorectal lesions, endoscopists can apply dyes and/or virtual chromoendoscopy [5]. Colorectal lesions are stained with dyes such as indigo carmine, crystal violet or methyl blue and are observed by colonoscopy with or without magnification. Virtual chromoendoscopy is an image-enhanced endoscopic technique based on optical strategies for capturing images. Currently, the most commonly used virtual chromoendoscopy systems in clinical practice are narrow-band imaging (NBI), flexible spectral imaging color enhancement (FICE), i-scan, and blue laser imaging (BLI) [5,6,7]. In NBI, optical filters are used to filter out red light and narrow the bandwidth to a range in which only blue and green light are visible [6]. Hemoglobin absorbs blue light, with a wavelength of 400–430 nm, to highlight the morphology of the surface capillaries and green light, with a wavelength of 525–555 nm, to penetrate and highlight deeper blood vessels. FICE and i-scan enable image capture for digital processing. In these methods, lesion images are enhanced and highlighted without being darkened like those in NBI. In the BLI system, lasers with a wavelength of 450 nm are used to irradiate phosphor and generate white light (WL), and blue lasers with a wavelength of 410 nm are employed for narrow-band image observations [7]. Blue light is absorbed by hemoglobin, thereby highlighting the morphology of the surface capillaries, and WL increases the brightness of the images to facilitate observations. Because the property of increased vascularity and change of blood vessel diameter in premalignant and malignant colorectal polyps, NBI is capable of identifying these lesions and predict the depth of invasion. Virtual chromoendoscopy has the advantage of rapid image conversion, without the influence of a time-consuming dyeing process and uneven dyeing. Virtual chromoendoscopy also prevents discomfort or cytotoxicity to the examinees caused by the dyes [6]. 

**Figure 1:**
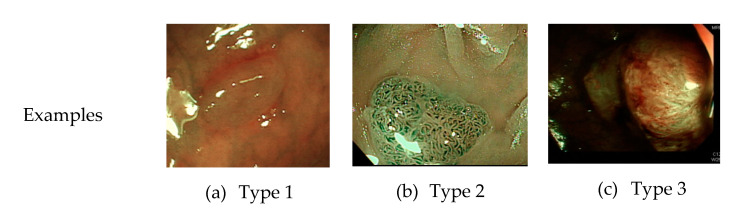
Example image of NICE classification.

Currently, common magnifying NBI diagnostic classifications are the Sano, Hiroshima, Showa, and Japan narrow-band imaging expert team (JNET) classifications, and the most prevalent non-magnifying NBI diagnostic classification is the NBI international colorectal endoscopic (NICE) classification (Table 1 and Figure 1) [8,9]. In most of these classifications, the histopathology of polyps is determined based on the optical characteristics of lesion color, structure of capillaries, and morphology of capillary surface. The NICE classification, for instance, categorizes colorectal polyps as type 1, hyperplastic polyps; type 2, adenomas, intramucosal carcinoma, or superficial submucosal carcinoma; and type 3, deep submucosal carcinoma. Rectosigmoid diminutive (≤5 mm) hyperplastic polyps are considered as non-neoplastic polyps without malignant potential [8]. The recently developed Workgroup serrAted polypS and Polyposis classification can enhance the diagnostic accuracy of sessile serrated adenoma polyps [10].

In 2011, the American Society of Gastrointestinal Endoscopy (ASGE) proposed a guideline for colorectal polyp diagnosis through a new optical endoscopic technique [11]. For diminutive polyps of the sigmoid colon and the rectum, the diagnose-and-leave or resect-and-discard strategy can be adopted if the correct diagnosis is made based on real-time endoscopic optics. The diagnose-and-leave strategy is applicable when the endoscopic optical diagnosis is highly precise (i.e., a negative predictive value of 90% and above for adenoma). Pathological biopsy is not required for such polyps. When the resect-and-discard strategy is adopted to remove diminutive colorectal polyps identified by high-confidence optical diagnosis, clinicians must combine optical diagnosis of rectosigmoid diminutive polyps with histopathological diagnosis for colorectal polyps >5 mm to determine the post-polypectomy surveillance interval. All colorectal polyps are sent for pathologic interpretation to ensure a consistency of 90%. Cost-effectiveness analyses have revealed that employing the innovative endoscopic optical technology to diagnose colorectal polyps and adopting either the diagnosis-and-leave or resect-and-discard strategy are cost-effective [12,13,14,15,16,17]. Nevertheless, the current challenge in the use of innovative endoscopic optical technology to diagnose colorectal polyps is that not all endoscopists meet the standards recommended by the ASGE. In fact, of the endoscopists in community hospitals, only 25% could achieved a colorectal polyp diagnosis accuracy of 90% by using the NBI-assisted optical diagnosis method [18], with the consistency rate of postoperative polyp resection monitoring intervals being only 85% and 81% [4,19]. Therefore, identifying the effective use of computer-aided diagnostic systems in helping clinicians decide the polyp treatment methods is imperative. 

Different computer-aided diagnostic systems for colonoscopy have been proposed [20,21,22]. The color, shape, texture, and spatial features of the images are analyzed through image processing techniques (e.g., noise removal, image sharpening, and image segmentation) and according to the optical characteristics captured using virtual chromoendoscopy (i.e., color of colorectal polyps, structure of capillaries, and morphology of capillary surface). The images are further classified or identified using grouping algorithms, and an identification accuracy and sensitivity of approximately 72–89% [4] and 90% [23], respectively. Through texture analysis and wavelet transformation, Hafner and Kwitt [23,24] computed the red-green-blue (RGB) and LAB models by using a color wavelet cross-cooccurrence matrix and analyzed the polyp classifications by applying K-nearest clustering and feature vectors. Gross et al. [25] employed nonlinear filters and Canny edge detectors for tumor contouring. Tamaki [26] extracted image feature vectors by using the scale-invariant feature transform and performed identification using support vector machine classification. However, determining a standard or single feature vector to classify different types of polyp images by using the said methods is difficult. 

In most computer-aided diagnostic systems, analysis is performed based on the magnification of endoscopic images. However, in clinical practice, non-magnified high-resolution colonoscopy images are most commonly used mainly because magnification endoscopes are more expensive, difficult to operate, and unsuitable for routine practice. Therefore, they are not preferred by general hospitals and clinicians and only used in medical centers for clinical research purposes. Several studies have proposed the application of deep learning to computer-aided diagnostic systems for colonoscopy, published data that conducted based on magnification endoscopic images yielded a diagnostic accuracy over 90% [21,27]. However, studies using non-magnification colonoscopic images for colorectal polyps classified by deep learning system is lacking. 

Deep learning has been increasing used in the medical field [28,29,30,31,32,33]. Many studies have applied a convolutional neural network (CNN) to the identification and classification of colorectal polyps [27,34]. Chen et al. [35] employed the deep neural network to differentiate between NBI images of neoplastic and hyperplastic polyps. Park et al. [36] used the CNN to classify the polyps in colonoscopy images. Ribeiro et al. [37] increased data sets through small patches and conducted polyp classification according to the features extracted using a CNN. Shin et al. [38] proposed the false-positive and offline learning method based on a region-based CNN (R-CNN), successfully improving the existing polyp locating method. By using Faster R-CNN, Ren et al. [39] developed a learning method by drawing a bounding box around a polyp. On the basis of the Faster R-CNN of the visual geometry group-16 model, Mo et al. [40] trained 16 colonoscopy images. Wang et al. [41] employed the SegNet model for examining colonoscopy images. Urban et al. [42] used VGG16 and VGG19 models to conduct analyses through 7-fold validation. Zheng et al. [43] used the you-only-look-once (YOLO) model to identify the polyp location in WL and NBI images. All these methods were adopted to locate polyps and assist clinicians for the classification of polyps. In most of these methods, training and testing were based on RGB images. However, because RGB images, expressed by three primary color layers, are multidimensional images, the computation cost involved is higher. In addition, the network frameworks were composed of a considerable number of layers, which makes the process time-consuming. 

**Figure 2:**
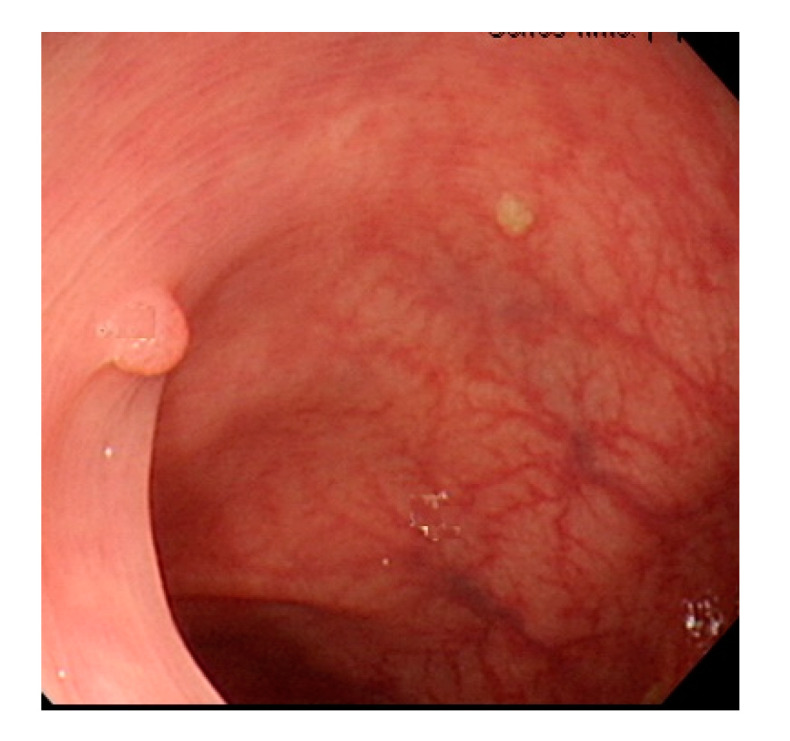
Excessive intestinal wall textures with small polyps.

**Figure 3:**
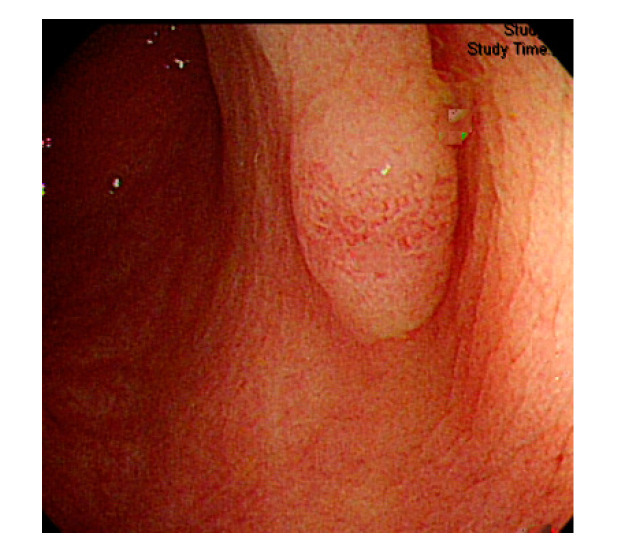
Small texture of polyp and intestinal wall.

Currently, many medical images are processed into grayscale images, such as ultrasound, computed tomography (CT), and magnetic resonance (MR) images. Tan et al. [44] applied a gray-level cooccurrence matrix and CNN to CT images for polyp diagnosis. Zhang et al. [45] compress the three-channel color images of chest CT to grayscale images. A five-layer deep CNN with stochastic pooling is used to diagnose chest-based COVID-19. Xie et al. [46] mentioned that in deep learning, colors are not the key features influencing accurate image classification. They also discovered that in X-ray image classification, the speed and accuracy of processing grayscale images were considerably higher than those of processing RGB images. The grayscale method used was ITU-R Recommendation BT.601 [47]. Moreover, misjudgments were easily made in the presence of excessive intestinal wall textures, when polyps were too small (Figure 2), and when polyp textures were similar (Figure 3) to intestinal wall textures.

In this study, we proposed a colorectal polyp detection and classification method by using grayscale images and a lightweight deep learning framework, which provided the same or similar detection effect as RGB images but incurred lower computing costs. To increase the applicability of the proposed method in clinical diagnosis, we developed a precise, computer-aided diagnostic system compatible with mobile devices. 

The rest of this study is organized as follows: Section 2 explains the materials and methods used in the colorectal polyp image detection and classification system; Section 3 details the experiments performed on the system, and Section 4 presents the discussion and conclusions.

## 2. Materials and Methods

**Figure 4:**
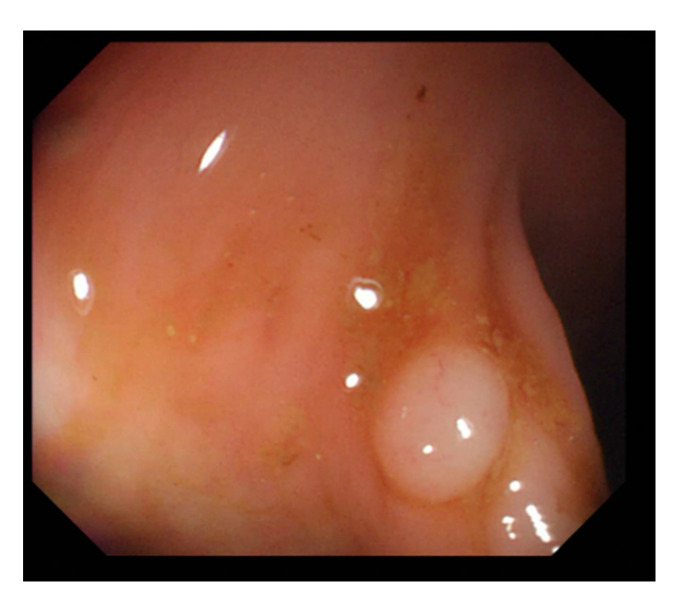
Colonoscopy image.

**Figure 5:**
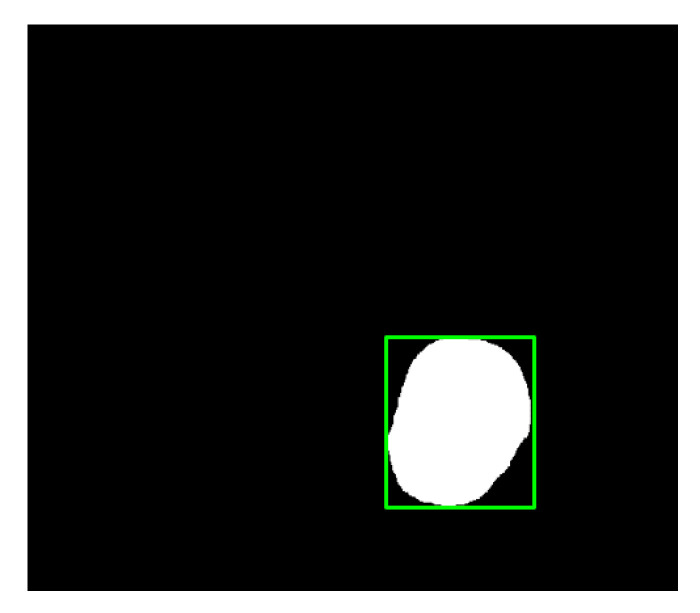
Segmented polyp image.

In this study, we employed colonoscopy images provided by the CVC Clinic [48,49] and the Department of Gastroenterology and Hepatology, Chang Gung Medical Hospital (CGMH) (Table 2). The CVC Clinic data set comprises 612 continuous images extracted from 29 WL images, among which 592 images had one polyp and the remaining 30 images had more than two polyps. The image resolution was 384 × 288 pixels. For image marking, the upper, lower, left, and right boundaries of the white part were set as the four sides of the bounding box according to the binarized ground truth (GT) obtained from the CVC Clinic data set. The endoscope models used were CF-260AI/AL and CF-290AI/AL (Olympus Optical Co, Ltd., Tokyo, Japan), and the endoscope host employed was EVIS LUCERA (Olympus Medical Systems) (Figure 4 and Figure 5). In both WL and NBI images, the parts with polyps were extracted. In all, 1000 polyp image data were captured, and 2160 image data were extracted from the experimental data, including 80 images of the same polyp from different angles. First, we selected one WL and one NBI image each from the 900 WL and 900 NBI colonoscopy images, respectively (1800 colonoscopy images in total). The image resolution was 640 × 480 pixels. Each image in the data set was manually marked by clinicians, and the file format used was tagged image file format (TIFF). In addition, we selected another 360 polyp images for a polyp classification test to improve classification accuracy.

**Figure 6:**
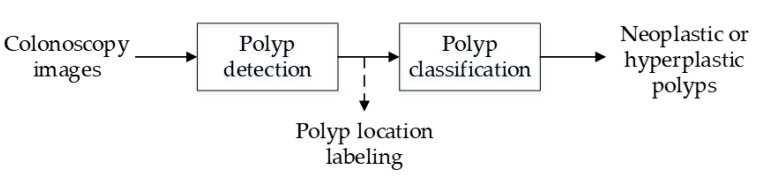
System architecture.

Figure 6 presents the architecture for the colorectal polyp detection and diagnosis system. The system is capable of polyp detection and classification. During colorectal polyp detection, the input colonoscopy images were preprocessed. The images in the data set were converted to grayscale images during preprocessing and applied for feature extraction and training to detect the location of polyps. The detected polyps were subsequently classified into neoplastic polyps (including adenomas and deep submucosal invasive cancer) and hyperplastic polyps.

### 2.1. Polyp Detection 

**Figure 7:**
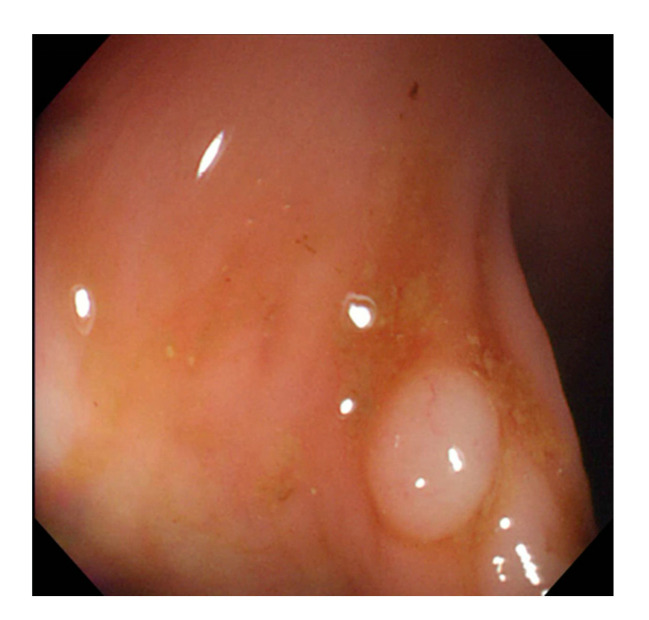
Polyp image before gray scaling.

**Figure 8:**
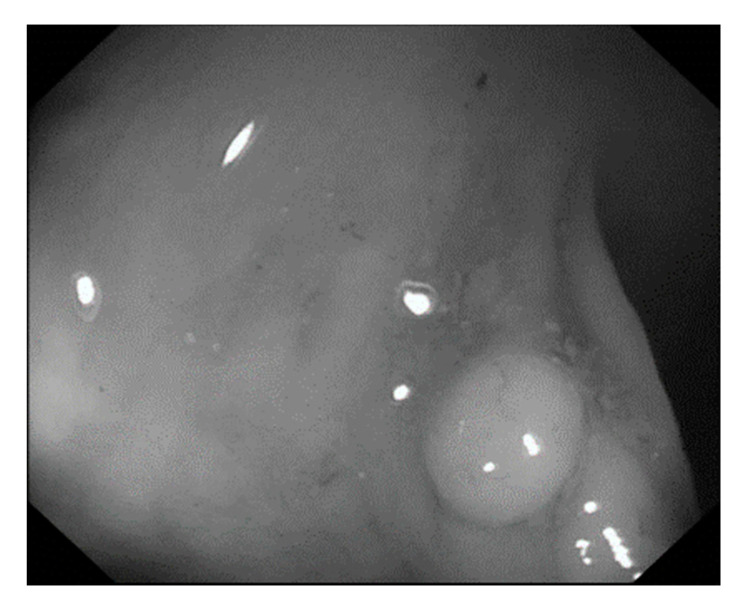
Polyp image after gray scaling.

The main polyp detection procedures involve the conversion of images to grayscale and labeling the location of polyps. During gray scaling, RGB images were converted to grayscale images with pixel values ranging from 0 to 255. RGB pixels were converted to gray pixels by using ITU-R BT.601 [47]:

Gray = 0.229 × R + 0.587 × G + 0.117 × B(1)

where R, G, and B are the pixel values of the red, green, and blue colors in color images, respectively (Figure 7 and Figure 8).

**Figure 9:**
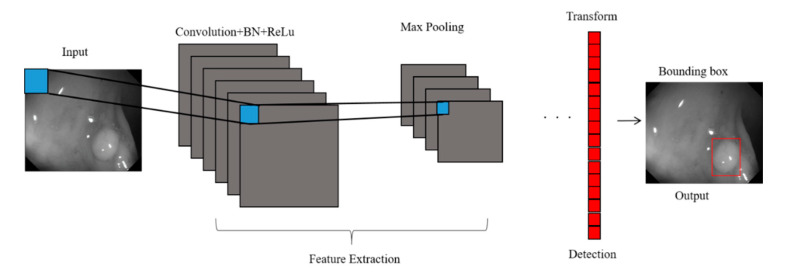
Colorectal polyp detection system architecture.

**Figure 10:**
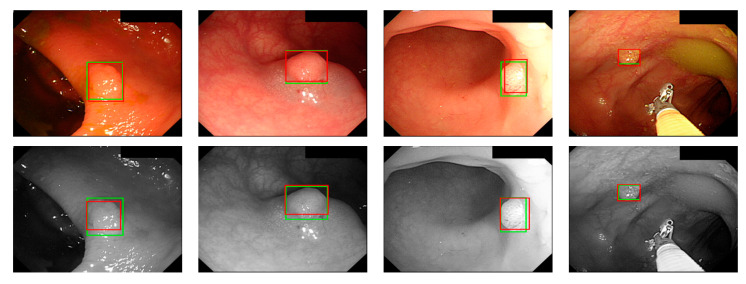
WL polyp detection.

**Figure 11:**
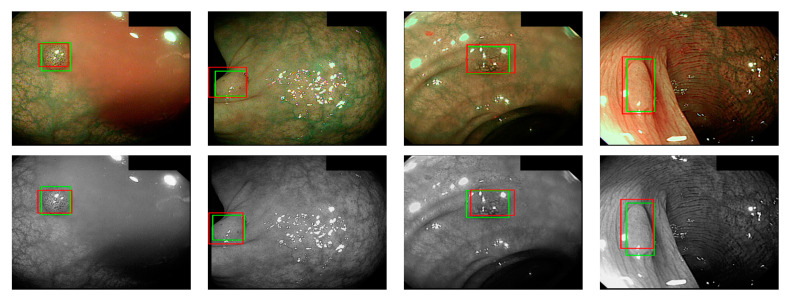
NBI polyp detection.

Furthermore, we adopted a CNN model for feature extraction (Figure 9). The analytical process included convolution, batch normalization (BN), rectified linear unit (ReLU) function implementation, and max pooling operations. The input colonoscopy images were scaled to 128 × 128 pixels, and a convolution filter was used for feature map extraction. The mean and standard deviation of the feature map were calculated in batches to normalize each channel of the input feature map. Subsequently, two trainable parameters, normalized through scaling and translation, were added to the model to effectively transfer the extracted feature map to the next layer, thereby increasing the training speed and enhancing the network stability. Threshold values of the normalized feature map that were less than 0 were set to 0. Through the filter, the maximum value of the feature map was obtained, thus highlighting the features:

(2) $$\mathrm{Feature}\;\mathrm{map}\left(x,y\right)=\underset{p}{\sum }\underset{q}{\sum }I\left(p,q\right)K\left(x-p+1,y-q+1\right)$$

where *I* is the input colonoscopy image; *K* denotes the filter; *p* and *q* are the horizontal and vertical axis coordinates of the original image, respectively; and *x* and *y* are the horizontal and vertical axis coordinates of the output feature map, respectively:

(3) $$\hat{x_{i}}=\frac{x_{i}-\mu_{B}}{\sqrt{\sigma_{B}^{2}+\varepsilon }}$$

(4) $$y_{i}=\gamma \hat{x_{i}}+\beta \equiv BN_{\gamma ,\beta }\left(x_{i}\right)$$

where $\hat{x_{i}}$ is the normalized output value; *x_i_* is the input value; *μ_B_* is the mean of the batches; *σ_B_*^2^ is the standard deviation of the batches; *ε* is the minimum value and *ε =* 10^−5^ in this study; *y_i_* is the output value of the normalized value; *γ* is the scaling parameter; and *β* is the translation parameter. The ReLU function performs threshold operations on the features in the upper layer, changing all values less than 0 to 0, which can clarify the features and facilitate network training. Threshold values of the normalized feature map that were less than 0 were set to 0:

(5) $$ReLU\left(BNy\right)=\{\begin{array}{c} \;BNy,BNy\geq 0\;\;\\ 0,BNy<0\; \end{array}$$

where *BNy* is the output value after *BN*, namely the input value of the *ReLU* function; The max pooling operation performs dimension reduction on the features to extract their maximum value. Through the filter, the maximum value of the feature map was obtained, thus highlighting the features:

(6) $$a_{j}={max}_{p}\left(a_{i}^{n\times n}\right)$$

where *a_i_* is the input value; *p* is the pooling regions; *n* is the pooling size; and *a_j_* is the output value after the maximum value is obtained. Polyp detection was then conducted in the bounding box transform layer. The prediction result of the feature map was obtained in the last layer and was output through a 1 × 1 convolutional layer by using a pixel-based approach and converted to a bounding box mode for display [50]. There are eight groups of bounding boxes to locate the polyps: (16, 16), (32, 32), (24, 48), (48, 24), (60, 80), (108, 72), (216, 144), and (180, 180). The cutoff value of polyp detection is 0.2. The size of the predicted bounding box was adjusted by converting it to the range of a true-value bounding box. Table 3 displays the framework of the CNN model. Figure 10 and Figure 11 show the results of WL and NBI polyp detection, respectively, where the first column is the RGB image; the second column is the grayscale image; the red box is the prediction box; and the green box is the actual polyp location marked by the clinician, namely the GT.

### 2.2. Polyp Classification 

According to the biopsy reports, we classified colorectal polyps into neoplastic polyps and hyperplastic polyps by using a CNN model (Table 4). The cutoff value of polyp classification is 0.5.

### 2.3. Statistical Analysis

Categorical data were presented as rates and proportions and 95% confidence intervals (CI) were calculated. The McNemar’s test was used to compare the accuracy of polyp detection between RGB and NBI images and grayscale images for deep learning system. A two-tailed *p* value less than 0.05 is considered statistically significant. The statistical analysis was performed by using IBM SPSS Statistics 22 (SPSS Inc., Chicago, IL, USA). 

## 3. Experimental Results

We divided the experiment data into three parts: the CVC Clinic images and the WL images and NBI images provided by the Chang Gung Medical Foundation. The images were divided based on data sets and different light sources to verify whether the dissimilarity and compatibility of images differed due to different light sources. The RGB and grayscale images in each data set were separately trained. This system was implemented using software, i.e., MATLAB version 2020b (The MathWorks, Inc., Natick, MA, USA).

### 3.1. Polyp Detection Results 

The data sets for polyp detection were divided into five groups (Table 5), and the data were input to the colorectal polyp detection system through a 5-fold cross validation for training and testing. During testing, calculations were performed based on the marked-up parts and the prediction box and according to the standard used in the 2015 endoscopic vision challenge held by the Medical Image Computing and Computer-Assisted Intervention Society (MICCAI-2015) [30,51]. Table 6, Table 7, Table 8, Table 9, Table 10, Table 11, Table 12, Table 13 and Table 14 presented the detection accuracy of our models for the tested data of the CGMH-WL, CGMH-NBI, and CGMH-WL+ CGMH-NBI images. The accuracy value of polyp detection is the number of correct detected polyp images divided by the total number of polyp images. The results indicated that the detection accuracy of grayscale images reached almost 95%, which was higher than of the RGB images when using WL image dataset (Table 6 and Table 7). However, there is no difference in the accuracy of polyp detection between NBI images and grayscale transformation images when using NBI image dataset (Table 8 and Table 9). Among RGB images, detection errors were noted in situations where polyp protrusions were nonobvious or had a texture similar to that of the intestinal wall, images had excessive noise, or the polyps were too small. Particularly, polyps with a size less than 1600 pixels (40 × 40 pixels) in the image were likely to be misjudged. Moreover, we conducted a deep learning for detection of polyp size divided into greater and smaller/equal than 0.5 cm (Table 12). The identification result of the gray scaled CNN exhibited no significant difference. The accuracy of the images with a polyp size both greater and smaller than 0.5 cm ranged between 94.4% and 96.5% (Table 13 and Table 14). That is, image size was the key factor affecting the detection accuracy. We used 900 normal colonoscopy images as the testing data to verify the performance of polyp detection. The accuracy of normal colonoscopy images detection was 870/900 = 96.56%. We found that some RGB polyp images that were not fully focused were likely to be undetected if the images were not transformed to grayscale. It is a common phenomenon in clinicians during colonoscopy screening and usually happened in the dynamic colonoscopy images. In addition, some unfocused NBI images may also be the possible cause of misjudgment for polyp detection.

### 3.2. Polyp Classification Results 

**Figure 12:**
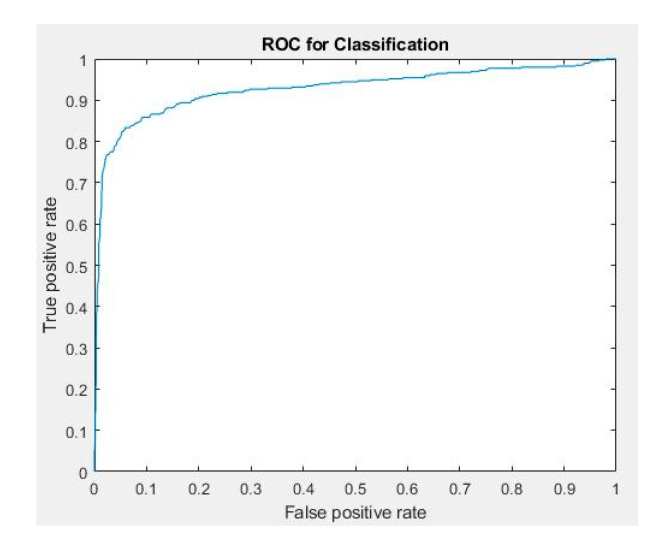
ROC curve for classification.

We classified 2072 WL and NBI colorectal polyp images provided by the Chang Gung Medical Hospital according to the results of pathological interpretation. The dataset was randomly split into training, validation, and testing group. None of the image shared between groups. The number of polyp images used for classification training, verification, and testing was 1370, 342, and 360, respectively (Table 15). The verification and testing data comprised the images that were accurately detected, and polyp images different from the preceding ones were used as the verification data. The 5-fold cross validation was also used for training, testing, and validation of polyp classification. Table 16 presents the performance index in which true positive (TP) is the situation where the center point of the prediction box falls at any GT; false positive (FP) is the situation where the center point of the prediction box does not fall at any GT; and false negative (FN) is the situation where no prediction box appears. True negative (TN) was not included because all the images in the data set had at least one polyp, implying that they were not negative images. Table 17 and Table 18 present the confusion matrices for the classification of WL and NBI grayscale images, respectively. Table 19 presents the performance evaluation of polyp classification. Figure 12 shows the ROC curve and the AUC value is 0.93. The results indicated that NBI images facilitated more accurate polyp identification than WL images. During polyp classification, neoplastic polyps were likely to be misjudged as hyperplastic polyps when polyp textures were not obvious, polyps were smaller than 1800 pixels, or when the images had an excessively low resolution. However, hyperplastic polyps were likely to be misjudged as neoplastic polyps because of the WL around polyps in the images and when polyps were smaller than 1800 pixels.

In Table 18, three neoplastic polyp images that were less than 1800 pixels were classified as hyperplastic polyps. The NPV for adenoma by using the proposed system is 84% (31/37), which did not reach the threshold of ≥90% for implementation of the diagnose-and-leave strategy. However, if the polyp images less than 1800 pixels were excluded from the study, the NPV is 91% (31/34), which achieved the threshold set by ASGE for diagnose-and-leave policy. 

## 4. Discussion and Conclusions

**Figure 13:**
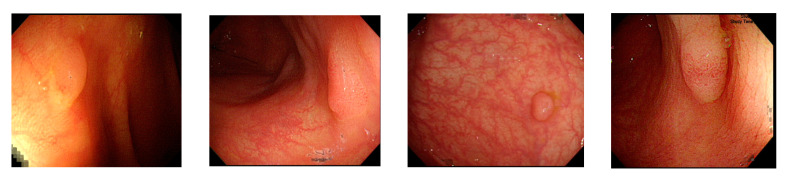
Nonobvious polyp protrusions or similar to intestinal wall.

**Figure 14:**
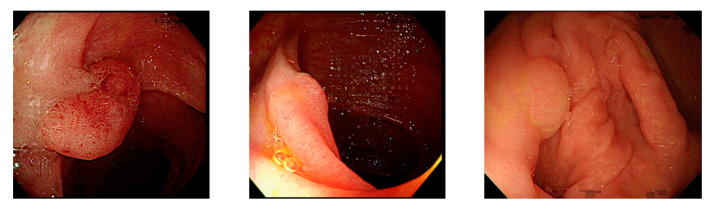
Excessive noise images.

**Figure 15:**
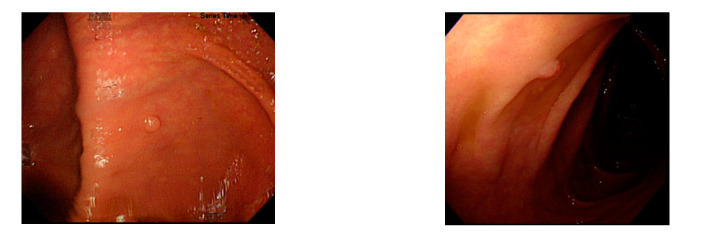
Small pixel size polyp images.

For polyp detection, the mean accuracy of grayscale images was 95.1%, which was higher than that of RGB/NBI images (94.1%). Detection errors in RGB images were caused when polyp protrusions were nonobvious or had a texture similar to that of the intestinal wall (Figure 13), images had excessive noise (Figure 14), or the polyps had an excessively small pixel size (Figure 15). Particularly, polyps with a size less than 1600 pixels (40 × 40 pixels) in the image were likely to be misjudged. Therefore, we recommend that in polyp detection, the length and width of polyp images must be at least 40 pixels in the lens regardless of the actual size of the polyps. This is likely to reduce misjudgment. We also found that when the colorectal polyps image is more complex, such as with more wrinkles or light spots, the use of color image deep learning for polyp detection may be more accurate. In addition, when the vascular lines of colorectal polyps were less obvious, such as unfocused or residual images, it would be better to use gray-scale image deep learning for polyp detection. Next step we plan to use a hybrid deep learning model that combines color and grayscale images for polyp detection in the dynamic colonoscopy videos. Maybe it can take the advantages of both methods as well as improve the detection accuracy.

**Figure 16:**
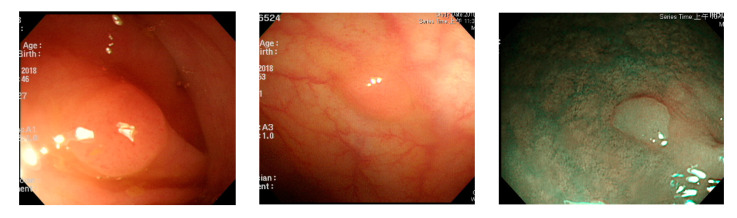
Unobvious polyp textures.

**Figure 17:**
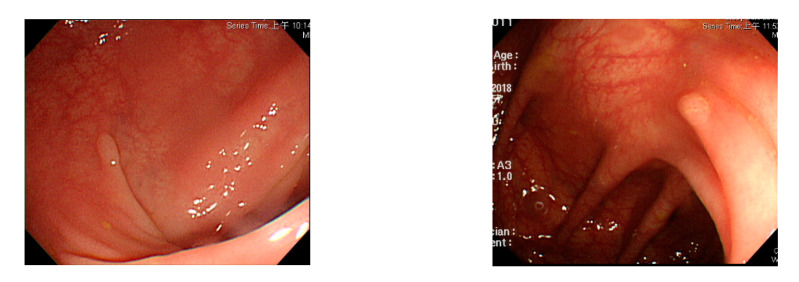
Polyp image size below 1800 pixels.

In polyp classification, neoplastic polyps were likely to be misjudged as hyperplastic polyps when polyp textures were not obvious (Figure 16), images had an excessively low resolution, or when polyps were smaller than 1800 pixels (Figure 17). Hyperplastic polyps, however, were likely to be misjudged as neoplastic polyps because of the WL around polyps in the images and when polyps were smaller than 1800 pixels.

In this study, we employed a deep learning framework for colorectal polyp detection by using grayscale images. The marked polyp data in the public database of the CVC Clinic and the clinical data provided by the Chang Gung Medical Hospital were collected for verification and testing. Rather than only using an analytic method for single-source data, we collected colonoscopy images captured by different brands and models of colonoscopes. This study had the following characteristics: first, the use of grayscaled RGB images facilitated the computation of the deep learning model. Second, in the deep learning framework, we used three convolutional filters (3 × 3, 1 × 3, and 3 × 1) in the convolutional layer to extract features. Different from conventional CNN models where single-feature filters were used, the proposed approach was capable of extracting more features for computation. We also compared the result with those obtained using other models and confirmed that the proposed approach could effectively increase the polyp detection accuracy. Third, grayscale images could reduce the dimensions and size of RGB images. Image deviation and noise caused by the difference in image gradient and endoscope lens could also be avoided, which increases the accuracy of polyp detection and classification. The results of the experiment indicate that in the detection of diminutive polyps—those smaller than 0.5 cm—the accuracy considerably decreased when polyps were smaller than 1600 pixels. The differentiation between neoplastic polyps and hyperplastic polyps became difficult when polyps were smaller than 1800 pixels. Hence, we recommend that clinicians could adjust the distance between the lens and polyps to enhance the detection results when conducting computer-assisted colorectal polyp analysis, which ensures that the polyps in the images have a minimum size of 1600 pixels.

Grayscale images achieve an equivalent or even higher accuracy of polyp detection than RGB/NBI images for lightweight computation. When NBI images were larger than 1800 pixels, our model could achieve high NPV (>90%) for neoplastic polyps. The colorectal polyp detection technique developed in this study can help clinicians rapidly locate polyps, whereas the colorectal polyp classification technique can assist clinicians in determining whether polypectomy or surgery is required, which may effectively improve patient outcome, avoid unnecessary polypectomy, and reduce misdiagnosis rate of endoscopists. In addition, hardware equipment is no longer limited by computation costs. The incorporation of simple mobile devices for edge computing and telemedicine services through lightweight computation and the hand-held colorectal polyp detection technique based on 5G wireless communication technology can substantially reduce the cost of medical equipment and increase medical standards.

## Figures and Tables

**Table 1:** NBI international colorectal endoscopic (NICE) classification.

Classification	Type 1	Type 2	Type 3
Color	Same or lighter than background	Browner than background	Brown to dark brown than background, sometimes patchy whiter areas
Vessels	None or isolated lacy	Brown vessels surrounding white structures	Areas of disrupted or missing vessels
Surface pattern	Dark or white spots of uniform size, or homogeneous absence of pattern	Oval, tubular or branched	Amorphous or abscent surface pattern
Diagnosis	Hyperplastic polyp	Adenoma	Deep submucosal invasive cancer

**Table 2:** Experiment data set.

Name	Image (N)	Resolution	Format
CVC-Clinic	612	384 × 288	.tif
CGMH-WL	1080	640 × 480	.tif
CGMH-NBI	1080	640 × 480	.tif

**Table 3:** Colorectal polyp detection model architecture.

Layers	Filters	Size/Stride	Output
Input			128 × 128
Convolutional	16	(3 × 3 + 3 × 1 + 1 × 3)/1	128 × 128
Batch Normalization			128 × 128
ReLu			128 × 128
Max pooling		2 × 2/2	64 × 64
Convolutional	32	(3 × 3 + 3 × 1 + 1 × 3)/1	64 × 64
Batch Normalization			64 × 64
ReLu			64 × 64
Max pooling		2×2/2	32 × 32
Convolutional	64	(3 × 3 + 3 × 1 + 1 × 3)/1	32 × 32
Batch Normalization			32 × 32
ReLu			32 × 32
Max pooling		2 × 2/2	16 × 16
Convolutional	128	(3 × 3 + 3 × 1 + 1 × 3)/1	16 × 16
Batch Normalization			16 × 16
ReLu			16 × 16
Convolutional	128	(3 × 3 + 3 × 1 + 1 × 3)/1	16 × 16
Batch Normalization			16 × 16
ReLu			16 × 16
Convolutional	128	(3 × 3 + 3 × 1 + 1 × 3)/1	16 × 16
Batch Normalization			16 × 16
ReLu			16 × 16
Convolutional	24	1 × 1/1	16 × 16
Transform			16 × 16
Output			

**Table 4:** Colorectal polyp classification model architecture.

Layers	Filters	Size/Stride	Output
Convolutional	16	(3 × 3 + 3 × 1 + 1 × 3)/1	480 × 640
Batch Normalization			480 × 640
ReLu			480 × 640
Max pooling		2 × 2/2	240 × 320
Convolutional	16	(3 × 3 + 3 × 1 + 1 × 3)/1	240 × 320
Batch Normalization			240 × 320
ReLu			240 × 320
Max pooling		2 × 2/2	120 × 160
Convolutional	32	(3 × 3 + 3 × 1 + 1 × 3)/1	120 × 160
Batch Normalization			120 × 160
ReLu			120 × 160
Max pooling		2 × 2/3	40 × 53
Convolutional	32	(3 × 3 + 3 × 1 + 1 × 3)/1	40 × 53
Batch Normalization			40 × 53
ReLu			40 × 53
Max pooling		2 × 2/3	13 × 18
Convolutional	64	(3 × 3 + 3 × 1 + 1 × 3)/1	13 × 18
Batch Normalization			13 × 18
ReLu			13 × 18
Max pooling		2 × 2/3	4 × 6
Convolutional	64	(3 × 3 + 3 × 1 + 1 × 3)/1	4 × 6
Batch Normalization			4 × 6
ReLu			4 × 6
Max pooling		2 × 2/1	2 × 3
Fully Connected			2
Softmax			

**Table 5:** Training and testing dataset of polyp detection.

Dataset	Training#	Testing#
CVC-Clinic	612	-
CGMH-WL	-	900
CGMH-NBI	-	900

**Table 6:** CGMH-WL testing dataset of polyp detection.

	RGB		Grayscale	
	Polyp	Normal	Polyp	Normal
Polyp	843	57	859	41
Accuracy% (95% CI)%	93.7 (91.9~95.2)		95.4 (93.9~96.7)	

**Table 7:** Comparison of polyp detection between RGB and grayscale images with CGMH-WLtesting dataset.

RGB	Grayscale		*p* Value
	Polyp Detected	No Polyp Detected	
Polyp detected	843	0	<0.001
No polyp detected	16	41	

**Table 8:** CGMH-NBI testing dataset of polyp detection.

	NBI		Grayscale	
	Polyp	Normal	Polyp	Normal
Polyp	850	50	853	47
Accuracy% (95% CI)%	94.4 (92.7~95.8)		94.8 (93.1~96.1)	

**Table 9:** Comparison of polyp detection between NBI and grayscale images with CGMH-NBI testing dataset.

NBI	Grayscale		*p* Value
	Polyp Detected	No Polyp Detected	
Polyp detected	850	0	0.25
No polyp detected	3	47	

**Table 10:** CGMH-WL + CGMH-NBI testing dataset of polyp detection.

	RGB + NBI		Grayscale	
	Polyp	Normal	Polyp	Normal
Polyp	1693	107	1712	88
Accuracy% (95% CI)%	94.1 (92.8~95.1)		95.1 (94.0~96.1)	

**Table 11:** Comparison of polyp detection between RGB + NBI and grayscale images with CGMH WL + CGMH-NBI testing dataset.

RGB + NBI	Grayscale		*p* Value
	Polyp Detected	No Polyp Detected	
Polyp detected	1693	0	<0.001
No polyp detected	19	88	

**Table 12:** The image number of polyps size greater and smaller than 0.5 cm.

	# of Polyp Size > 0.5 cm	# of Polyp Size ≤ 0.5 cm	Subtotal
WL	313	587	900
NBI	294	606	900
Total	607	1193	1800

**Table 13:** Polyp > 0.5 cm.

	WL		NBI	
	Polyp	Normal	Polyp	Normal
Polyp	302	11	281	13
Accuracy% (95% CI)%	96.5 (93.8~98.2)		95.6 (92.6~97.6)	

**Table 14:** Polyp size ≤ 0.5 cm.

	WL		NBI	
	Polyp	Normal	Polyp	Normal
Polyp	557	30	572	34
Accuracy% (95% CI)%	94.9 (92.8~96.5)		94.4 (92.2~96.1)	

**Table 15:** Polyp classification dataset.

Data Type	Neoplastic Polyps	Hyperplastic Polyps	Subtotal	Percentage
Training#	685	685	1370	1370/1712 = 80%
Verification#	263	79	342	342/1712 = 20%
Subtotal	856	856	1712	100%
Testing#	248	112	360	360/360 = 100%
(Training#) + (Verification#) + (Testing#) = 2072 images				

**Table 16:** Performance indices.

Accuracy (Acc)	$\mathrm{Acc}=\frac{\mathrm{TP}+\mathrm{FN}}{\mathrm{TP}+\mathrm{FP}+\mathrm{TN}+\mathrm{FN}}$
Precision (Prec)	$\mathrm{Prec}=\frac{\mathrm{TP}}{\mathrm{TP}+\mathrm{FP}}$
Recall (Rec)	$\mathrm{Rec}=\frac{\mathrm{TP}}{\mathrm{TP}+\mathrm{FN}}$
F1-measure (F1)	$F1=\frac{2\times \mathrm{Prec}\times \mathrm{Rec}}{\mathrm{Prec}+\mathrm{Rec}}$
F2-measure (F2)	$F2=\frac{5\times \mathrm{Prec}\times \mathrm{Rec}}{4\times \mathrm{Prec}+\mathrm{Rec}}$

**Table 17:** Confusion matrix of WL polyp classification.

	Neoplastic (Predicted)	Hyperplastic (Predicted)
Neoplastic (Actual)	110	14
Hyperplastic (Actual)	36	20

**Table 18:** Confusion matrix of NBI polyp classification.

	Neoplastic (Predicted)	Hyperplastic (Predicted)
Neoplastic(Actual)	118	6
Hyperplastic(Actual)	25	31

**Table 19:** Performance index of polyp classification.

	Acc%	Prec%	Rec%	F1%	F2%
WL	72.2	75.3	88.7	81.5	85.7
NBI	82.8	82.5	95.2	88.4	92.3

## References

[1] Sung H., Ferlay J., Siegel R.L., Laversanne M., Soerjomataram I., Jemal A., Bray F. (2021). Global Cancer Statistics 2020: GLO-BOCAN Estimates of Incidence and Mortality Worldwide for 36 Cancers in 185 Countries. CA Cancer J. Clin..

[2] Zauber A.G., Winawer S.J., O’Brien M.J., Ballegooijen M., Hankey B.F. (2012). Colonoscopic polypectomy and long-term pre-vention of colorectal-cancer deaths. N. Engl. J. Med..

[3] Suzuki H., Yamamura T., Nakamura M., Hsu C.M., Su M.Y., Chen T.H., Chiu C.T., Hirooka Y., Goto H. (2020). An International Study on the Diagnostic Accuracy of the Japan Narrow-Band Imaging Expert Team Classification for Colorectal Polyps Ob-served with Blue Laser Imaging. Digestion.

[4] Kuiper T., Marsman W.A., Jansen J.M., van Soest E.J., Haan Y.C., Bakker G.J., Fockens P., Dekker E. (2012). Accuracy for Optical Diagnosis of Small Colorectal Polyps in Nonacademic Settings. Clin. Gastroenterol. Hepatol..

[5] Subramanian V., Ragunath K. (2014). Advanced Endoscopic Imaging: A Review of Commercially Available Technologies. Clin. Gastroenterol. Hepatol..

[6] Kaltenbach T., Sano Y., Friedland S., Soetikno R., American Gastroenterological A. (2008). American Gastroenterological Association (AGA) Institute technology assessment on image-enhanced endoscopy. Gastroenterology.

[7] Yoshida N., Hisabe T., Inada Y., Kugai M., Yagi N., Hirai F., Yao K., Matsui T., Iwashita A., Kato M. (2013). The ability of a novel blue laser imaging system for the diagnosis of invasion depth of colorectal neoplasms. J. Gastroenterol..

[8] Hewett D., Kaltenbach T., Sano Y., Tanaka S., Saunders B.P., Ponchon T., Soetikno R., Rex D.K. (2012). Validation of a Simple Classification System for Endoscopic Diagnosis of Small Colorectal Polyps Using Narrow-Band Imaging. Gastroenterology.

[9] Hayashi N., Tanaka S., Hewett D.G., Kaltenbach T.R., Sano Y., Ponchon T., Saunders B.P., Rex D.K., Soetikno R.M. (2013). Endoscopic prediction of deep submucosal invasive carcinoma: Validation of the narrow-band imaging international colo-rectal endoscopic (NICE) classification. Gastrointest. Endosc..

[10] IJspeert J.E., Bastiaansen B.A., van Leerdam M.E., Meijer G.A., van Eeden S., Sanduleanu S., Schoon E.J., Bisseling T.M., Spaander M.C., van Lelyveld N. (2016). Development and validation of the WASP classification system for optical diagnosis of adenomas, hyperplastic polyps and sessile serrated adenomas/polyps. Gut.

[11] Rex D.K., Kahi C., O’Brien M.J., Levin T., Pohl H., Rastogi A., Burgart L., Imperiale T., Ladabaum U., Cohen J. (2011). The American Society for Gastrointestinal Endoscopy PIVI (Preservation and Incorporation of Valuable Endoscopic Innovations) on real-time endoscopic assessment of the histology of diminutive colorectal polyps. Gastrointest. Endosc..

[12] Lieberman D., Moravec M., Holub J., Michaels L., Eisen G. (2008). Polyp Size and Advanced Histology in Patients Undergoing Colonoscopy Screening: Implications for CT Colonography. Gastroenterology.

[13] Chiu H.-M., Chang L.-C., Shun C.-T., Wu M.-S., Wang H.-P. (2014). Current management of diminutive colorectal polyps in Taiwan. Dig. Endosc..

[14] Kang Y.K. (2014). Diminutive and Small Colorectal Polyps: The Pathologist’s Perspective. Clin. Endosc..

[15] Abu Dayyeh B.K., Thosani N., Konda V., Wallace M.B., Rex D.K., Chauhan S.S., Hwang J.H., Komanduri S., Manfredi M., Maple J.T. (2015). ASGE Technology Committee systematic review and meta-analysis assessing the ASGE PIVI thresholds for adopting real-time endoscopic assessment of the histology of diminutive colorectal polyps. Gastrointest. Endosc..

[16] Hassan C., Pickhardt P.J., Rex D.K. (2010). A Resect and Discard Strategy Would Improve Cost-Effectiveness of Colorectal Cancer Screening. Clin. Gastroenterol. Hepatol..

[17] Kessler W.R., Imperiale T., Klein R.W., Wielage R.C., Rex D.K. (2011). A quantitative assessment of the risks and cost savings of forgoing histologic examination of diminutive polyps. Laryngo-Rhino-Otol..

[18] Ladabaum U., Fioritto A., Mitani A., Desai M., Kim J.P., Rex D.K., Imperiale T., Gunaratnam N. (2013). Real-Time Optical Biopsy of Colon Polyps with Narrow Band Imaging in Community Practice Does Not Yet Meet Key Thresholds for Clinical Decisions. Gastroenterology.

[19] Paggi S., Rondonotti E., Amato A., Terruzzi V., Imperiali G., Mandelli G., Terreni N., Lenoci N., Spinzi G., Radaelli F. (2012). Resect and discard strategy in clinical practice: A prospective cohort study. Endoscopy.

[20] Mori Y., Kudo S.E., Berzin T.M., Misawa M., Takeda K. (2017). Computer-aided diagnosis for colonoscopy. Endoscopy.

[21] Takemura Y., Yoshida S., Tanaka S., Kawase R., Onji K., Oka S., Tamaki T., Raytchev B., Kaneda K., Yoshihara M. (2012). Computer-aided system for predicting the histology of colorectal tumors by using narrow-band imaging magnifying colonoscopy (with video). Gastrointest. Endosc..

[22] Kominami Y., Yoshida S., Tanaka S., Sanomura Y., Hirakawa T., Raytchev B., Tamaki T., Koide T., Kaneda K., Chayama K. (2015). Computer-aided diagnosis of colorectal polyp histology by using a real-time image recognition system and narrow-band imaging magnifying colonoscopy. Gastrointest. Endosc..

[23] Hafner M., Brunauer L., Payer H., Resch R., Wrba F., Gangl A., V’ecsei A., Uhl A. Pit Pattern Classification of Zoom-endoscopical Colon Images using DCT and FFT. Proceedings of the IEEE International Symposium on Computer-Based Medical Systems.

[24] Häfner M., Kwitt R., Uhl A., Wrba F., Gangl A., Vécsei A. (2009). Computer-assisted pit-pattern classification in different wavelet domains for supporting dignity assessment of colonic polyps. Pattern Recognit..

[25] Gross S., Kennel M., Stehle T., Wulff J., Tischendorf J., Trautwein C., Aach T., Meinzer H.P., Handels H., Tolxdorff T. (2013). Polyp Segmentation in NBI Colonoscopy. Bildverarbeitung für die Medizin.

[26] Tamaki T., Yoshimuta J., Takeda T., Raytchev B., Kaneda K., Yoshida S., Takemura Y., Tanaka S. (2010). A System for Colorectal Tumor Classification in Magnifying Endoscopic NBI Images. Proceedings of the 10th Asian Conference on Computer Vision.

[27] Korbar B., Olofson A.M., Miraflor A.P., Nicka C.M., Suriawinata M.A., Torresani L., Suriawinata A.A., Hassanpour S. Looking Under the Hood: Deep Neural Network Visualization to Interpret Whole-Slide Image Analysis Outcomes for Col-orectal Polyps. Proceedings of the 2017 IEEE Conference on Computer Vision and Pattern Recognition Workshops (CVPRW).

[28] Liedlgruber M., Uhl A. (2011). Computer-Aided Decision Support Systems for Endoscopy in the Gastrointestinal Tract: A Review. IEEE Rev. Biomed. Eng..

[29] Suzuki K. (2017). Overview of deep learning in medical imaging. Radiol. Phys. Technol..

[30] Shen D., Wu G., Suk H.-I. (2017). Deep Learning in Medical Image Analysis. Annu. Rev. Biomed. Eng..

[31] Lu L., Zheng Y., Carneiro G., Yang L. (2017). Deep Learning and Convolutional Neural Networks for Medical Image Computing. Precision Medicine, High Performance and Large-Scale Datasets.

[32] Litjens G., Kooi T., Bejnordi B.E., Setio A.A.A., Ciompi F., Ghafoorian M., van der Laak J.A., van Ginneken B., Sánchez C.I. (2017). A survey on deep learning in medical image analysis. Med. Image Anal..

[33] Zhang Y.-D., Satapathy S.C., Guttery D.S., Górriz J.M., Wang S.-H. (2020). Improved Breast Cancer Classification Through Combining Graph Convolutional Network and Convolutional Neural Network. Inf. Process. Manag..

[34] Liu Q. (2017). Deep Learning Applied to Automatic Polyp Detection in Colonoscopy Images.

[35] Chen P.-J., Lin M.-C., Lai M.-J., Lin J.-C., Lu H.H.-S., Tseng V.S. (2018). Accurate Classification of Diminutive Colorectal Polyps Using Computer-Aided Analysis. Gastroenterology.

[36] Park S.Y. Colonoscopic polyp detection using convolutional neural networks. Proceedings of the SPIE 9785, Medical Imaging 2016: Computer-Aided Diagnosis.

[37] Ribeiro E., Uhl A., Hafner M. Colonic Polyp Classification with Convolutional Neural Networks. Proceedings of the 2016 IEEE 29th Inter-national Symposium on Computer-Based Medical Systems (CBMS).

[38] Shin Y., Qadir H.A., Aabakken L., Bergsland J., Balasingham I. (2018). Automatic Colon Polyp Detection Using Region Based Deep CNN and Post Learning Approaches. IEEE Access.

[39] Ren S., He K., Girshick R., Sun R. (2015). Faster R-CNN: Towards Real-Time Object Detection with Region Proposal Networks. Adv. Neural Inf. Process. Syst..

[40] Mo X., Tao K., Wang Q., Wang G. (2018). An Efficient Approach for Polyps Detection in Endoscopic Videos Based on Faster R-CNN. Proceedings of the 24th IEEE International Conference on Pattern Recognition (ICPR).

[41] Wang P., Xiao X., Glissen Brown J.R., Berzin T.M., Tu M., Xiong F., Hu X., Liu P., Song Y., Zhang D. (2018). Development and validation of a deep-learning algorithm for the detection of polyps during colon-oscopy. Nat. Biomed. Eng..

[42] Urban G., Tripathi P., Alkayali T., Mittal M., Jalali F., Karnes W., Baldi P. (2018). Deep Learning Localizes and Identifies Polyps in Real Time With 96% Accuracy in Screening Colonoscopy. Gastroenterology.

[43] Zheng Y., Yu R., Jiang Y., Mak T.W.C., Wong S.H., Lau J.Y.W., Poon C.C.Y. Localisation of Colorectal Polyps by Convolutional Neural Network Features Learnt from White Light and Narrow Band Endoscopic Images of Multiple Data-bases. Proceedings of the 40th Annual International Conference of the IEEE Engineering in Medicine and Biology Society.

[44] Tan J., Gao Y., Cao W., Pomeroy M., Zhang S., Huo Y., Li L., Liang Z. GLCM-CNN: Gray Level Co-occurrence Matrix based CNN Model for Polyp Diagnosis. Proceedings of the 2019 IEEE EMBS International Conference on Biomedical & Health Informatics (BHI).

[45] Zhang Y.-D., Satapathy S.C., Liu S., Li G.-R. (2020). A five-layer deep convolutional neural network with stochastic pooling for chest CT-based COVID-19 diagnosis. Mach. Vis. Appl..

[46] Xie Y., Richmond D. Pre-training on Grayscale ImageNet Improves Medical Image Classification. Proceedings of the Computer Vision–ECCV 2018 Workshops.

[47] ITU ITU-R Recommendations Retrieved BT.601. https://www.itu.int/rec/R-REC-BT.601/.

[48] Bernal J., Sánchez J., Vilariño F. (2012). Towards automatic polyp detection with a polyp appearance model. Pattern Recognit..

[49] Bernal J., Sánchez F.J., Fernández-Esparrach M.G., Gil D., Rodríguez C., Vilariño F. (2015). WM-DOVA maps for accurate polyp highlighting in colonoscopy: Validation vs. saliency maps from physicians. Comput. Med Imaging Graph..

[50] Redmon J., Farhadi A. YOLO9000: Better, Faster, Stronger. Proceedings of the 2017 IEEE Conference on Computer Vision and Pattern Recognition (CVPR).

[51] Bernal J., Tajkbaksh N., Sánchez F.J., Matuszewski B.J., Chen H., Yu L., Angermann Q., Romain O., Rustad B., Balasingham I. (2017). Comparative Validation of Polyp Detection Methods in Video Colonoscopy: Results from the MICCAI 2015 Endoscopic Vision Challenge. IEEE Trans. Med. Imaging.

